# Decolonising primary health care practice: a definition and its importance

**DOI:** 10.5694/mja2.52683

**Published:** 2025-07-07

**Authors:** Tamara J Mackean, Kim O'Donnell, Juanita Sherwood, Shane D'Angelo, Madison Shakespeare, Cleone Wellington, Toby Freeman, Anna M Ziersch, Matt Fisher, Deborah A Askew, Judith M Dwyer, Annette J Browne, Fran Baum AO

**Affiliations:** ^1^ Flinders University Adelaide SA; ^2^ University of Technology Sydney Sydney NSW; ^3^ Waminda Nowra NSW; ^4^ Stretton Institute, University of Adelaide Adelaide SA; ^5^ University of Queensland Brisbane QLD; ^6^ University of British Columbia Vancouver Canada

**Keywords:** Social determinants of health, Community health services, Delivery of healthcare, Health services, Primary health care, Indigenous health

Decolonising primary health care (PHC) is essential to improving Aboriginal and Torres Strait Islander peoples’ health and wellbeing. Having a definition of decolonising PHC provides a valuable resource for creating and maintaining culturally safe practices within health policy and health services. We developed such a definition through collaboration between Aboriginal researchers, non‐Indigenous researchers, PHC services, health professionals and sector stakeholders. Our definition provides a robust basis for (re)building trust with Aboriginal and Torres Strait Islander people through explicit acknowledgement of colonising harms and explicit ways in which these harms can be mitigated. The CONSIDER reporting criteria checklist for health research involving Indigenous peoples^1^ was completed for this article and can be found in the Supporting Information.

Australia is, and has been since time immemorial, under the care and custodianship of Aboriginal and Torres Strait Islander Nations.^2^ The British invasion in 1788 and subsequent occupation through its colonial rule and governance, legislation and policy, illegally took control through force with no negotiation or treaty.^3^ Australians continue to occupy contested lands and seas. The violence of colonisation is extreme and ongoing, with the last known massacre of Aboriginal peoples in 1928.^4^ Aboriginal and Torres Strait Islander peoples have been targeted by successive colonist governments resulting in premature deaths, dislocation from lands, suppression of culture and silencing of leaders and advocates.^5^, ^6^ Children continue to be stolen from their families through a public service system that remains steeped in its colonial roots.^7^, ^8^ The imposition of the colony has disastrous impacts on the health and wellbeing of Aboriginal and Torres Strait Islander peoples.^9^ Health systems and services are complicit in the imposition of colonial values and are often found to be racist and inaccessible to Aboriginal and Torres Strait Islander peoples.^10^, ^11^ Accordingly, a process of decolonisation is vital to ensure that the health system does not continue to perpetuate harm from colonial attitudes and behaviours, including the exclusion of Aboriginal and Torres Strait Islander knowledges and practices regarding health and healing.^10^, ^12^ This perspective article seeks to answer the question: how do Aboriginal and Torres Strait Islander PHC services define decolonising practice?

## Positionality statement

The author group is a mixture of Aboriginal and non‐Indigenous researchers across all career stages. TM is a Waljen woman and public health medicine physician; KO is a Malyangapa/Barkindji woman and public health researcher; JS is a Wiradjuri woman and decolonising health researcher; SD is a Kokotha man and Indigenous health researcher; MS is a First Nations Australian and interdisciplinary researcher; CW is a Cullughutti/Jerrinja/Wandi‐Wandian woman and member of the chief executive leadership team of Waminda; TF is a non‐Aboriginal man and health equity researcher; AZ is a non‐Aboriginal woman and public health social scientist; MF is a non‐Aboriginal man and senior research fellow in public health; DA is a non‐Aboriginal woman and primary health care researcher; JD is a non‐Aboriginal woman and health services researcher; AB is a non‐Indigenous woman and health inequities researcher; and FB is a non‐Aboriginal woman and community health and commercial and social determinants of health researcher.

## Approach to developing the definition

Our definition was developed as part of the National Health and Medical Research Council (GNT1139348) Decolonising Primary Health Care Project, 2018–2024, which was approved by Aboriginal health ethics committees (Aboriginal Health Research Ethics Committee, South Australia [04‐19‐809 | 04‐20‐904], and Aboriginal Health & Medical Research Council Human Research Ethics Committee [1538/19 | 1744/20]) and health system ethics committees (Central Australian Human Research Ethics Committee, Human Research Ethics Committee of Northern Territory Department of Health and Menzies School of Health Research [2021‐33995], Southern Adelaide Clinical Human Research Ethics Committee, Metro South Health Human Research Ethics Committee [HREC/19/QMS/54681]). The research design for the overall project is based on cooperative inquiry with five Aboriginal and Torres Strait Islander PHC services (both community‐controlled and mainstream services) as research partners and a Project Advisory Group (PAG) of key stakeholders comprising representatives of peak Aboriginal and Torres Strait Islander health sector organisations.^13^ Over the course of the project (2019 to 2023), we conducted 68 semi‐structured interviews and 14 workshops with staff at partner PHC services, eight community forums with users of those services and 20 yarns with PHC stakeholders working in key government and non‐government organisations.^14^

The definition built on earlier work of some of our team, which applied cultural safety concepts to public policy development processes,^15^ existing literature on decolonisation^16^, ^17^ and through researcher, service partner and PAG discussion and consideration of emerging findings, existing wisdom and significant lived experience.

Yarning^18^ was used as an overarching methodology for both data collection, data analysis and meaning making. Dadirri, or deep listening,^19^ of excerpts of interview and workshop recordings during research meetings allowed the whole team to collectively identify critical elements of decolonising practice. The Aboriginal research team met separately on a regular basis (2022–2023) for discussions about research conduct and data analysis, which were then fed back to the wider team. Input and feedback were received from PHC service partners and the PAG through tabling and discussion at meetings (2020–2023). The definition was revised iteratively in light of feedback until consensus was reached. This is in line with decolonising approaches that uphold Indigenous ways of knowing (through reflection and prior knowledge), being (through relationship and collaboration) and doing (through iteration and consensus).^20^

## Definition of decolonising PHC practice

Although there is existing literature on the importance of decolonisation,^12^ there has not been an accurate definition of decolonising practice for the Australian PHC sector. Our definition has been developed to apply to PHC as a site for health promotion, prevention, early intervention, advocacy and action to influence upstream colonising health determinants. These are core functions of PHC and are increasingly critical for health system sustainability given the finite resources available.^21^ Decolonisation is not a single event or single process and it looks different across diverse First Nations and their sovereign Countries. Our definition is as follows:

“Colonisation in Australia is built as a structure of systemic racism and contributes to major effects on the health and wellbeing of Aboriginal and Torres Strait Islander peoples. Decolonising primary health care practices are the ways of working that unpack colonial approaches to health, so Aboriginal and Torres Strait Islander peoples can not only survive but thrive. This means transforming the policies, processes and practices that influenced health in the past, and which are still present today.

Decolonising practice:

- is led by Aboriginal and Torres Strait Islander ways of knowing, being and doing;
- breaks down systemic racism;
- challenges the power imbalances in all structures formed by society;
- acknowledges and addresses white privilege; and
- is strengths‐based.”

## Practical uses of the definition

Decolonisation is not an easy concept for health services and staff to understand. It goes beyond anti‐racism and cultural safety, which all services should be pursuing. Decolonising practice looks very different in different settings, and the way colonisation is unpacked through decolonising processes will require different strategies for state, territory and national legislations, policies and systems.

Appreciating the value of decolonising practice requires an understanding of the history of the white settler colony and the severe implications this created and continues to create for Aboriginal and Torres Strait Islander peoples in Australia. It also requires an understanding of the importance of self‐determination to health and wellbeing. The “No” result from the Voice to Parliament referendum held in October 2023^22^ suggests that many Australians do not understand these links, despite the health benefits being supported by evidence.^23^ Having a definition that reflects this history, and its impact provides a valuable resource for health professional training, advancing best practice and challenging deficit discourse in health policy. Institutions such as universities and medical colleges can incorporate the definition into curricula to build the knowledge, skills and attitudes of practitioners to ensure adverse implicit biases and power imbalances are able to be identified and mitigated in health care interactions.^24^

For Aboriginal and Torres Strait Islander community‐controlled and government health services, a clear definition will facilitate the confrontation of white privilege and reveal unhelpful colonial structures. The aim is to guide non‐Indigenous staff to take responsibility for addressing and decolonising their understandings and approaches to Aboriginal and Torres Strait Islander health. Health services themselves need to develop processes that will enable any assumptions of privilege and colonising ways of working to be managed constructively, not evaded or denied.^10^ Adopting our collaboratively developed definition of decolonising practice will provide a strong foundation from which to introduce the notion of decolonisation and an effective starting point for reform of organisational policies and practices, including staff orientation and ongoing professional development. The definition situates decolonisation as vital to understanding the historical and current context of colonial policy and the long shadow it casts over current health service delivery.^16^ Disrupting unsafe practice and resetting norms from deficit to strength is not a combative process, indeed, these actions require critical thinking and reflexivity. Without this contextual understanding and reflexive abilities, the provision of culturally safe services by non‐Indigenous peoples is highly unlikely.^24^, ^25^

For Aboriginal and Torres Strait Islander staff, the definition sets out clearly what decolonising practice is, which can be empowering. The definition provides the opportunity for truth‐telling, inclusivity and reconciliation.^2^ For example, one of our service partners provides parenting support for Aboriginal and Torres Strait Islander parents. At our community workshop, families involved in the program all agreed that the anguish of the Stolen Generations was a barrier to becoming involved in the service. They had observed that children are still being removed by government agencies and the effect has been multigenerational and continuing as the report of the Human Rights and Equal Opportunity Commission^26^ demonstrates. A definition of decolonising practice provides a robust basis for (re)building trust with Aboriginal and Torres Strait Islander people through explicit acknowledgement of harms, and potential harms, and explicit ways in which these harms can be mitigated. Increasing trust means Aboriginal and Torres Strait Islander people are more likely to use health services rather than avoid them.^10^, ^23^

## Conclusion

Adoption of this definition of decolonising PHC practice developed by PHC services and stakeholders can lead to health and wellbeing benefits for Aboriginal and Torres Strait Islander peoples. The definition offers benefits derived from an unambiguous understanding of the harms of colonisation. It also provides a strong basis for the advancement of culturally safe health services and a pathway to the much‐needed health system transformation that is required for health equity in Australia. Our work was limited to our service partners regarding examples of decolonising PHC and other services may have provided different information and reflections. Future research could examine the breadth of decolonising practice within Australian PHC and provide further evidence for system change.

## Open access

Open access publishing facilitated by Flinders University, as part of the Wiley – Flinders University agreement via the Council of Australian University Librarians.

## Competing interests

No relevant disclosures.

## Provenance

Not commissioned; externally peer reviewed.

## Author contribution statement

Mackean T: Conceptualization, formal analysis, funding acquisition, investigation, methodology, project administration, supervision, writing – original draft, writing – review and editing. O’Donnell K: Formal analysis, investigation, methodology, writing – review and editing. Sherwood J: Formal analysis, investigation, methodology, writing – review and editing. D’Angelo S: Formal analysis, investigation, writing – review and editing. Shakespeare M: Formal analysis, investigation, writing – review and editing. Wellington C: Conceptualization, investigation, project administration, writing – review and editing. Freeman T: Conceptualization, data curation, formal analysis, funding acquisition, investigation, methodology, project administration, supervision, writing – original draft, writing – review and editing. Ziersch A: Conceptualization, formal analysis, funding acquisition, investigation, methodology, project administration, supervision, writing – review and editing. Fisher M: Data curation, formal analysis, investigation, writing – review and editing. Askew D: Project administration, writing – review and editing. Dwyer J: Conceptualization, funding acquisition, writing – review and editing. Browne A: Conceptualization, funding acquisition, writing – review and editing. Baum F: Conceptualization, data curation, formal analysis, funding acquisition, investigation, methodology, project administration, supervision, writing – review and editing.

## Supporting information


CONSIDER statement
