# Supplementary material for: Decolonising primary health care practice: a definition and its importance
Source: Med J Aust. 2025 Jul 7;223(1):9–12. doi: 10.5694/mja2.52683 (PMC12230911; doi:10.5694/mja2.52683)
Supplement: Supplementary file 1 — CONSIDER statement [file MJA2-223-9-s001.pdf]

Supporting Information

Supplementary material

This appendix was part of the submitted manuscript and has been peer reviewed.
It is posted as supplied by the authors.

Appendix to: Mackean TJ, O'Donnell K, Sherwood J, et al. Decolonising primary health care practice: a definition and its importance. *Med J Aust* 2025; doi: 10.5694/mja2.52683.

CONSIDER Statement: Decolonising primary health care practice: a definition and its importance

Guest Editors of the 2025 *Indigenous Health Special Issue* acknowledge the Indigenous expertise that informed the establishment of the CONSolIDated critERia for strengthening the reporting of health research involving Indigenous Peoples (CONSIDER) statement.

Authors should indicate how they have supported ethical publishing and reporting practices by providing the details of the research practices aligned with this publication in accordance with the CONSIDER statement. The reporting should not exceed two pages. This reporting will be published as online supplementary information. Detailed items can be accessed in the publication:

https://bmcmedresmethodol.biomedcentral.com/articles/10.1186/s12874-019-0815-8

Governance
The Decolonising Primary Health Care (DPHC) project developed relationships with the PHC service partners and with stakeholders through the Project Advisory Group (PAG). Representatives from the service partners were associate investigators on the grant application and provided input into methodology, aims and objectives. The PAG included representatives of peak Aboriginal and Torres Strait Islander health sector organisations. Service partners and PAG provided feedback on the conduct of the project over the course of the project via research meetings and workshops.
**Prioritization**
The project utilised cooperative inquiry with service partners contributing to research questions developed to reflect the priorities of the entire group. Three service partners were Aboriginal community-controlled primary health care services and two were mainstream primary health care services for Aboriginal and Torres Strait Islander people. A range of methods (including interviews, yarning, community forums, staff workshops, deep listening and logic modelling) that enabled different contexts to be examined was also deemed important.
**Relationships (Indigenous stakeholders/participants and Research Team**
The project involved data collection in four states and territories, and ethics approvals were received from appropriate bodies in each jurisdiction (seven in total), including the Aboriginal Health Research Ethics Committee in South Australia where the administering institution for the grant was located.<br>The research team included six Aboriginal researchers across all stages of career with expertise in decolonisation, community engagement and Indigenous research methodologies and methods.
**Methodologies**
The research designed was based on cooperative inquiry and utilised yarning and deep listening methods alongside qualitative methods for data collection and analysis as well as overall meaning making. Separate meetings of Aboriginal and non-Aboriginal researchers provided opportunities for different contexts and power dynamics to be discussed safely. Group sessions on deep listening of excerpts of recorded interviews and workshops allowed for diverse insights into decolonising practice, which were also shared at PAG meetings.
**Participation**
The project was affected by the coronavirus disease 2019 pandemic and aspects of the research that involved in-person participation had to be delayed. Notwithstanding these delays, the project used a range of data collection and analysis methods that enabled participation by community members (through community forums), stakeholders (through interview and yarns) and service partner staff (through interviews and workshops). Data collected was stored on password-protected servers at the administering institution and access to the data required authorisation from the lead researchers.
**Capacity**
Aboriginal researchers involved in the project had the opportunity to lead academic papers (as first author) as well as contribute to academic papers (as co-authors). Service partners were also invited to be co-authors on outputs of interest to them. Separate meetings of non-Indigenous researchers enabled engagement with the concept of “imperfect allies” and the appropriate role of non-Indigenous researchers in decolonising research. Separate meetings of the Aboriginal researchers enabled culturally safe spaces for discussion of whiteness, privilege and racism.

Analysis and interpretation

Aboriginal researchers led the development of a code book used for analysis of yarning and interview data with the specific intent of having a strengths-based approach to analysis and interpretation. Thematic analysis occurred under overarching themes of lived experience/narratives; context and power; health and wellbeing; social/political barriers and facilitators; and organisational practices. In addition, group sessions on deep listening of excerpts of recorded interviews and workshops allowed for diverse insights into decolonising practice from a variety of perspectives, which added to the robustness of findings and implications.

Dissemination

A range of academic papers were developed to communicate findings. In addition, a community report was produced that provided an overview of the project in plain English. In response to service partner advocacy, a short film was produced for community and other services to enable engagement with findings in an audio-visual way rather than as text. Several conference presentations were developed and delivered jointly by Aboriginal and non-Indigenous researchers, including researchers from service partners.
